# Post-Translational Modifications and Diabetes

**DOI:** 10.3390/biom14030310

**Published:** 2024-03-06

**Authors:** Chiranjeev Sharma, Abu Hamza, Emily Boyle, Dickson Donu, Yana Cen

**Affiliations:** 1 Department of Medicinal Chemistry, Virginia Commonwealth University, Richmond, VA 23219, USA; sharmac3@vcu.edu (C.S.); hamzaa@vcu.edu (A.H.); boyleel2@vcu.edu (E.B.); donud@vcu.edu (D.D.); 2 Institute for Structural Biology, Drug Discovery and Development, Virginia Commonwealth University, Richmond, VA 23219, USA

**Keywords:** post-translational modifications, diabetes, T1D, T2D, clinical trials

## Abstract

Diabetes and its associated complications have increasingly become major challenges for global healthcare. The current therapeutic strategies involve insulin replacement therapy for type 1 diabetes (T1D) and small-molecule drugs for type 2 diabetes (T2D). Despite these advances, the complex nature of diabetes necessitates innovative clinical interventions for effective treatment and complication prevention. Accumulative evidence suggests that protein post-translational modifications (PTMs), including glycosylation, phosphorylation, acetylation, and SUMOylation, play important roles in diabetes and its pathological consequences. Therefore, the investigation of these PTMs not only sheds important light on the mechanistic regulation of diabetes but also opens new avenues for targeted therapies. Here, we offer a comprehensive overview of the role of several PTMs in diabetes, focusing on the most recent advances in understanding their functions and regulatory mechanisms. Additionally, we summarize the pharmacological interventions targeting PTMs that have advanced into clinical trials for the treatment of diabetes. Current challenges and future perspectives are also provided.

## 1. Introduction

Diabetes mellitus is a chronic metabolic disorder that directly impacted 537 million people worldwide, causing 6.7 million deaths in 2021 [1]. It also poses a huge economic burden to the global healthcare systems. Diabetes is caused when the body is unable to regulate blood sugar properly either due to impaired insulin secretion or sensitivity. Genetic and environmental factors can also contribute to its development [2]. Traditionally, diabetes can be classified into two types: type 1 diabetes (T1D) and type 2 diabetes (T2D). Diabetes that begins during pregnancy is called gestational diabetes. A state of high blood glucose level that is not high enough to be considered T2D is called prediabetes [3]. Both gestational diabetes and prediabetes can advance into T1D or T2D [4]. While T1D is an autoimmune disorder that needs continuous insulin replacement therapy, T2D can be prevented by dietary and lifestyle changes [5]. Regular exercise can help in reducing the potential risk of developing this disorder. Unfortunately, such simple lifestyle and dietary changes can be a daunting task for a diabetic patient. Thus, it creates an immediate need for alternative approaches to contain it. Extensive research is ongoing to understand the pathophysiology and underlying molecular mechanism causing the onset and progression of diabetes [6]. It is a field of active investigation.

Post-translational modifications (PTMs) of proteins are chemical or enzymatic processes by which translated proteins can be reversibly or irreversibly covalently modified [7]. These modifications, such as glycosylation, glycation, *O*-GlcNAcylation, AMPylation, citrullination, deamidation, carbonylation, methylation, oxidation, SUMOylation, and so forth, can affect the stability, localization, and function of proteins, consequently influencing several cellular processes, in turn interfering with the normal functioning of the cell [8]. Pertinent literature suggests that these PTMs play a vital role in the modulation of several cellular responses, such as immune and inflammatory responses, which influence the molecular mechanisms relevant to diabetes [9,10].

Some PTMs are observed at the onset of diabetes mellitus. The PTM known as AMPylation or adenylylation is a reversible covalent modification that involves transferring an adenosine monophosphate (AMP) moiety from ATP onto a hydroxylated side-chain amino acid on the protein [11]. This modification impacts the Hsp70 chaperone protein called BiP [12]. AMPylation leads to a specific conformation of BiP with reduced substrate affinity and impaired responsiveness to co-factors that typically stimulate its activity, thus deactivating the chaperone function [13]. AMPylation of BiP is also important for the endoplasmic reticulum (ER) proteostasis network. Mutations in the FICD gene, which encodes an enzyme responsible for regulating BiP AMPylation, is closely related to infancy-onset diabetes mellitus [14]. It has been demonstrated that cells with mutated FICD genes exhibit elevated levels of AMPylated BiP, in turn interfering with proper ER functions and compromising insulin secretion. These findings not only highlight the critical role of BiP in maintaining normal physiology but may also guide the development of potential antidiabetic therapeutic strategies targeting FICD. Such interventions hold promise in alleviating the adverse consequences of infancy-onset diabetes. Moreover, in a diabetic pregnancy, maternal metabolism is altered, leading to the formation of reactive α-dicarbonyls in the reproductive organs and embryos. The enzyme glyoxalase (GLO) 1 is known to detoxify the reactive α-dicarbonyls and safeguard against modifications of proteins by advanced glycated end products (AGEs). It was shown that maternal diabetes affects the activity of GLO1 by post-translational modification due to changes in the metabolic processes in the embryos [15]. Better understanding of the potential roles of PTMs in diabetes is providing insights into the molecular mechanisms that can be targeted to develop antidiabetic drugs [16].

The classical analytical approaches used in the study of protein PTMs include mass spectrometry (MS)-based proteomic analysis [17] and PTM-specific antibody recognition [18]. MS enables the extensive, precise, and large-scale profiling of PTMs with high sensitivity [19]. Antibody-based techniques including Western blotting, immunohistochemistry, and protein microarray are also popular and considered efficient methods for PTM analysis.

**Figure 1:**
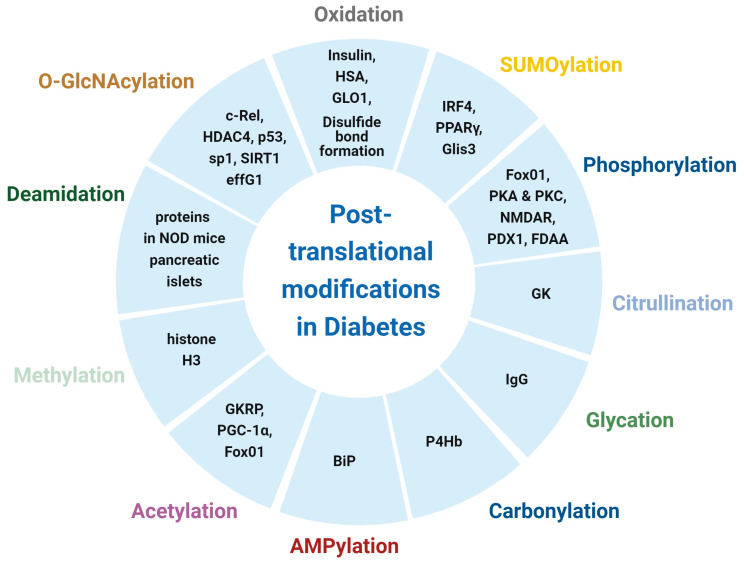
PTMs in diabetes. The figure was created with BioRender.com.

In this review, we endeavour to shed light on the advancement of the research related to the PTMs in T1D and T2D within the last five years (Figure 1). We also provide an overview of clinical trials of diabetes by small molecules that target PTMs. Comprehensive investigation of individual PTM, as well as PTM crosstalk, may provide potential therapeutic strategies to reverse diabetes-related pathways.

## 2. PTMs in Type 1 Diabetes

T1D is an insulin-dependent autoimmune disorder. It comprises up to 10% of the total diabetic population and usually develops in children and young adults. Impaired functioning of insulin-producing β-cell leads to the development of T1D [20]. Numerous PTMs have been identified as autoimmune biomarkers that play important roles in the onset and progression of T1D. There are several reports suggesting the role of PTM in T1D, as shown in Table 1.

### 2.1. Oxidation

Insulin therapy is at the forefront of T1D management [33]. PTMs of insulin can also increase autoimmune reactions in diabetes. Strollo et al. reported antibodies specific to oxidatively post-translationally modified insulin (oxPTM-INS, insulin modified by hydroxyl radical) as a novel biomarker for improved prediction of T1D in children with positive standard islet autoantibodies [21]. Oxidative PTM has also been used in studying the initiation and progression of T1D. Onset of diabetes is strongly correlated with oxidative stress. Oxidative stress generates reactive oxygen species (ROS), which are also associated with AGEs [34]. Both ROS and AGEs can impair β-cell function, advancing to T1D development. Hyperglycemia induces oxido-nitrosative stress in T1D, producing immunologically active human serum albumin (HSA), presumably through glycation-induced oxidation [22].

### 2.2. Glycation and N-Glycosylation

Hyperglycemia also leads to extensive glycation of immunoglobulins (IgG and IgM) in T1D [23]. Upon glycation, the molecular architecture of immunoglobulins is significantly changed compared to unmodified immunoglobulins, resulting in decreased reactivity. While glycation is non-enzymatic, glycosylation is an enzyme-mediated PTM. Significant changes in plasma *N*-glycosylation have been observed in children at the onset of T1D [24]. This study enables the development of a predictive model incorporating glycan data, which have immense potential in T1D risk assessment. Furthermore, marked changes of *N*-glycosylation on human complement C3 have been detected in children with early-onset T1D [25], underpinning the potential of the C3 *N*-glycan profile as a risk factor in T1D.

### 2.3. Carbonylation

Carbonylation is an irreversible PTM associated with the early onset of T1D autoimmunity. In response to inflammation and oxidative stress, carbonylation occurs to introduce reactive carbonyl moieties into the protein structures. Carbonylation has been detected in pancreatic islets before the onset of hyperglycemia in mice [26]. Carbonylation of the β subunit of prolyl-4-hydroxylase (P4Hb), a multifunctional enzyme responsible for folding and trafficking of proinsulin, is amplified in pancreatic islets, resulting in an increased proinsulin-to-insulin ratio [26]. This is followed by an upsurge of anti-insulin autoantibodies and β-cell dysfunction in T1D. It has been suggested that antioxidant therapeutics may alter carbonylation and can be a useful strategy in treating T1D.

### 2.4. Citrullination

Citrullination is the deimination of arginine to citrulline catalyzed by calcium-dependent peptidylarginine deiminases (PADs) [35]. The citrullination of pancreatic glucokinase (GK) as a result of inflammation further triggers autoimmunity and influences GK activity to suppress insulin secretion [27]. GK regulates glycogen synthesis in hepatocytes and operates as a glucose sensor in pancreatic b cells [36]. GK citrullination reduces islet function and impairs glucose homeostasis. Pharmacological inhibition of PAD2/PAD4 partially restores the compromised insulin secretion. The positive feedback loop of proinflammatory cytokines and citrullination contributes significantly to T1D pathology [27].

### 2.5. Deamidation

Deamidation is PTM catalyzed by transglutaminases (TGMs) to convert glutamine to glutamic acid [37]. The expression and activity of TGM2 in nonobese diabetic (NOD) mice were examined in [28]. While TGM2 was upregulated in the pancreatic islets of the NOD mice, it was downregulated in the medullary thymic epithelial cells (mTECs). Furthermore, TGM2 expression can be stimulated by inflammatory cytokines such as IL1b and IFNg [28]. Together, these results underscore the importance of TGM2 in generating deamidated autoantigens in T1D.

### 2.6. O-GlyNAcylation

*O*-GlcNAcylation involves the covalent attachment of a single *N*-acetyl-β-D-glucosamine (*O*-GlcNAc) to the hydroxyl group of serine or threonine residues. It regulates multiple cellular processes, including immune response and autoimmunity. The transcriptional regulation of immunosuppressive forkhead box P3 (FOXP3) by c-Rel *O*-GlcNAcylation has been reported recently [29]. c-Rel, a member of the NF-kB family, controls the expression of genes responsible for T-cell activation and function [38]. Hyperglycemia is shown to increase c-Rel *O*-GlcNAcylation. This PTM, at Ser350 in particular, reduces the binding affinity of c-Rel in the FOXP3 promoter region to suppress FOXP3 transcription in mouse models of autoimmune diabetes [38].

The production of a cardio-protective *N*-terminal fragment of histone deacetylase 4 (HDAC4) is upregulated in diabetic patients due to enhanced HDAC4 *O*-GlcNAcylation at Ser642 [30]. This PTM further impedes Ca^2+^/calmodulin-dependent protein kinase II (CaMKII)-mediated phosphorylation at the adjacent Ser632, a cardio-detrimental PTM [30]. These results suggest an intricate regulatory interplay between distinct PTMs.

### 2.7. SUMOylation

The reversible covalent attachment of small ubiquitin-related modifier (SUMO) proteins, or SUMOylation, is closely related to T1D pathogenesis. Ubc9 is the only mammalian E2 conjugating enzyme involved in the SUMOylation process [39]. Macrophage-specific deletion of Ubc9 has been shown to attenuate macrophage energy homeostasis and M2-type polarization, exacerbating the risk of streptozotocin (STZ)-induced T1D [31]. At the molecular level, Ubc9-catalyzed SUMOylation of interferon regulator factor 4 (IRF4) promotes its nuclear localization, stability, and transcriptional activity to enhance the M2 program [31]. Therefore, pharmacological modulation of aberrant SUMOylation in macrophages has been suggested as a potential therapeutic intervention in T1D.

### 2.8. Methylation

Diabetes-induced histone PTMs have profound consequences in the pathology of diabetic complications [40]. Decreased expression of glucose transporter GLUT4, encoded by *Slc2a4* gene, in the skeletal muscle of T1D- and T2D-like animals has been detected [32]. Mechanistically, diabetes-induced H3K9me3 in the promoter region of *Slc2a4* suppresses its expression, thereby contributing to glycemic impairment [32]. Targeting histone PTMs may open new avenues for the development of novel therapeutics for diabetes.

## 3. PTMs in Type 2 Diabetes

Insulin resistance and malfunctioning of β-cells are regarded as the primary physiological factors leading to type 2 diabetes (T2D). Research findings have indicated that the first factor arises from issues with insulin signaling, problems with glucose transport, and the negative effects of excess lipids. On the other hand, the second factor, β-cell dysfunction, could potentially be linked to the accumulation of amyloid in islets, oxidative stress (OS), an overabundance of fatty acids, and the absence of incretin influences [41]. In the following section, we discuss the role of different PTMs in T2D (Table 2).

### 3.1. O-GlcNAcylation

Earlier studies suggested that elevated *O*-GlcNAcylation levels resulting from increased expression of *O*-GlcNAc transferase (OGT) or inhibition of *O*-GlcNAcase (OGA) might contribute to insulin resistance through the hexosamine biosynthetic pathway (HBP) [62,63]. For example, the *O*-GlcNAcylation of insulin receptor substrate 1 (IRS-1) hindered the functioning of AKT, leading to the onset of insulin resistance [64]. Furthermore, it has been demonstrated that *O*-GlcNAcylation of PDK1 and AKT attenuate the insulin signaling pathway [65,66]. *O*-GlcNAcylation of glycogen synthase (GS) was also found to be a contributing factor to the decreased enzyme activation observed in cases of insulin resistance [67].

Hepatic p53 plays an important role in maintaining glucose homeostasis. It is stabilized by *O*-GlcNAcylation upon fasting. Subsequently, it activates the transcription of phosphoenolpyruvate carboxykinase 1 (PCK1) to regulate glucose production [42]. In the liver of individuals with T2D, increased expressions of p53 and the proteins associated with *O*-GlcNAcylation, such as OGT and glutamine-fructose-6-phosphate amidotransferase (GFAT), have been observed [42]. These changes were thought to be closely associated with insulin insensitivity and hyperglycemia in T2D patients [42]. The presence of glucose in the blood also led to elevated *O*-GlcNAcylation of specificity protein 1 (Sp1), causing a reduction in the expression of Sp1-dependent Pi-class Glutathione *S*-transferase (GTSP), an innate defense mechanism against reactive oxygen species (ROS) [43]. This decrease has been suggested to contribute to the development of oxidative stress, a characteristic of the diabetic condition.

SIRT1 is an NAD^+^-dependent protein deacetylase that has been implicated in the regulation of hepatic gene expression [68,69,70]. The *O*-GlcNAcylation of SIRT1 has been suggested to control its interactions with other proteins and its spatiotemporal dynamics in a nutrient-dependent manner [44]. During the transition from fasting to re-feeding, the *O*-GlcNAcylation of SIRT1 within the N-terminal domain switches its interactions with transcription factors to AKT, a cytosolic kinase that is closely related to insulin signaling. Sustained *O*-GlcNAcylation also causes a cellular translocalization of SIRT1 from the nucleus to cytosol for further ubiquitin-mediated degradation, further strengthening the notion that this particular PTM offers spatiotemporal control of SIRT1 functions [44]. In contrast, hypoglycosylation of SIRT1 upon nutrient deprivation enhances its interactions with transcription factors, resulting in abnormal gene expression, impaired mitochondrial function, increased glucose production in the liver, and, ultimately, a diabetes-like state [44].

Insufficient processing of insulin before secretion, or hyperproinsulinemia, is considered an early hallmark of T2D [71,72]. Hyperproinsulinemia has been observed in β cell-specific OGT knockout (βOGTKO) mice [45]. A reduction in carboxypeptidase E (CPE), a key insulin processing enzyme, in the islets of these mice is also detected, partially due to the decreased translation caused by a loss of eukaryotic translation initiation factor 4γ1 (eIF4G1) [45]. The stability of eIF4G1 depends on its O-GlcNAcylation status at Ser61. Thus, the OGT-eIF4G1-CPE axis seems to dictate insulin processing in β cells. This is further supported by the improved CPE levels and reversed hyperproinsulinemia upon eIF4G1 overexpression [45].

OGT-regulated β-cell O-GlcNAcylation has also been suggested to link hyperlipidemia to β-cell functions in the prediabetic phase [46]. Partial deletion of β-cell OGT compromises the lipid potentiation of insulin secretion [46]. On the molecular level, sarco/ER Ca^2+^ ATPase 2 (SERCA2) has been identified as an endogenous β-cell OGT target. *O*-GlcNAcylation at a particular threonine residue has been proposed to regulate SERCA2 function through Ca^2+^ binding. Pharmacological activation of SERCA 2 by a small-molecule allosteric activator restores lipid-stimulated insulin secretion [46].

Excessive visceral fat is closely associated with insulin resistance and other metabolic risk factors [73]. High levels of *O*-GlcNAcylation in visceral adipose tissue have been observed during fasting as a mechanism to retain fat mass [47]. Reduced *O*-GlcNAcylation and enhanced phosphorylation of perilipin 1 (PLIN1) caused by adipose OGT deletion result in lipolysis stimulation. On the contrary, overexpression of adipose OGT inhibits fat breakdown in the adipose tissue and triggers diet-induced obesity, leading to whole-body insulin resistance.

### 3.2. Phosphorylation

It has long been postulated that aberrant phosphorylation of islet proteins is critical for the initiation and development of T2D [74]. However, the underlying molecular mechanism remains elusive. Using a combination of proteomic and phosphoproteomic approaches, the significant remodeling of key signaling pathways in T2D islets has been revealed [48]. For example, marked dephosphorylation of protein kinase A (PKA) and protein kinase C (PKC) substrates, along with decreased expression levels of the catalytic subunits of PKA and PKC, has been observed in *db*/*db* islets, all of which lead to hyperglycemia. Additionally, glycogen synthase kinase 3 (GSK3) has been identified as a key player in regulating β-cell-specific PDX1, a transcription factor essential for glucose sensing and insulin secretion [48,75]. Hyperactivation of GSK3 in *db*/*db* islets results in the phosphorylation and proteasome degradation of PDX1 and subsequently contributes to the impairment of β-cell functions [48].

Brown adipocyte (BA) has been suggested as a determinant in the progression of metabolic disorders [76,77]. The activation of BAs is known to restore insulin sensitivity and reduce blood glucose in T2D patients [78,79]. In a recent study, a substantial reduction in the expression of protein tyrosine phosphatase receptor type B (PTPRB) was observed during brown adipocyte differentiation [49]. The dephosphorylation of vascular endothelial growth factor receptor 2 (VEGFR2) caused by the overexpression of the wild-type but not the catalytically inactive PTPRB suppresses brown adipocyte differentiation, presumably through the downregulation of brown adipogenic genes [49]. Thus, PTPRB has been suggested as a negative regulator of brown adipocyte differentiation and a potential therapeutic target for the treatment of T2D.

Adipocyte-secreted leptin is a long-term biochemical signal regulating caloric homeostasis [80]. Targeting the leptin receptors in the hypothalamus region, leptin plays key roles in improving insulin sensitivity and fatty acid oxidation and decreasing triacylglycerol synthesis [81]. Additionally, leptin has been found to trigger the activation of Src kinase for the subsequent phosphorylation of the GluN2A subunit of the *N*-methyl-D-aspartate receptor (NMDAR), primarily in the pancreatic b cells [50]. This phosphorylation enhances NMDAR currents and promotes the translocation of ATP-sensitive potassium channels (K_ATP_) and Kv2.1 channels to the cell membrane, ultimately resulting in b-cell membrane hyperpolarization. The decreased excitability of b cells leads to a reduction in glucose-mediated insulin secretion. This hypothesis was further supported by the observation that leptin failed to stimulate membrane hyperpolarization in b cells from leptin-resistant *db*/*db* mice and obese diabetic patients [50].

Metformin is the first-line treatment for T2D [82]. The effects of metformin hydrochloride and metformin glycinate have been compared [83]. Both formulations demonstrate comparable antihyperglycemic effects in mouse and human hepatocytes. However, higher levels of phosphorylated AMPK and its substrate, acetyl-CoA carboxylase (ACC), have been detected in metformin glycinate-treated hepatocytes. Metformin glycinate administration also enhances the phosphorylation of AKT, at Ser473 in particular, for improved insulin sensitivity in hepatocytes from *db*/*db* mice or humans [83].

Forkhead box protein O1 (FoxO1) is a transcription factor that has been implicated in metabolism regulation and T2D pathology [84]. Specifically, insulin-promoted PI3K/AKT activation is known to phosphorylate FoxO1 and enhance its cytoplasmic retention [85,86]. On the contrary, insulin resistance compromises PI3K/AKT signaling to enhance FoxO1 nuclear retention and transcriptional activity [84]. The phosphorylation status of FoxO1 is also under the control of progestin and adipoQ receptor 3 (PAQR3), a Golgi membrane protein [51]. In palmitic acid (PA)-induced insulin-resistant HepG2 cells, the upregulation of PAQR3 has been observed. This overexpression leads to decreased levels of phosphorylated FoxO1, GK, and low-density lipoprotein receptor (LDLR) [51]. PA stimulation also promotes the nuclear accumulation of p65 and its physical interactions with FoxO1, suggesting the potential involvement of PAQR3 in activating FoxO1 through the NF-kB pathway in insulin resistance.

Fas-associated death domain-containing protein (FADD) is known to regulate insulin secretion and islet development [87]. FADD phosphorylation has been suggested to be involved in insulin degradation, as evidenced by the compromised insulin production and increased insulin levels in serum of FADD-D (FADD phosphorylation-mimic mutation) mice [87]. A recent study demonstrated that FADD phosphorylation downregulates the mRNA and protein levels of insulin-degrading enzymes (IDEs) [52]. At the transcription level, FADD phosphorylation promotes the nuclear translocation of FoxO1 to suppress IDE transcription. At the protein level, this PTM decreases IDE protein stability. Overall, the study suggested a new role of FADD in regulating IDE, although the underlying molecular mechanism awaits further investigation.

Maintaining ER homeostasis is essential for normal β-cell function. ER stress disrupts ER homeostasis and subsequently activates unfolded protein response (UPR) signaling [88,89]. Protein kinase R-like endoplasmic reticulum kinase (PERK), a component of the UPR pathway, serves as an ER stress sensor to phosphorylate the α subunit of eukaryotic translation initiation factor 2 (eIF2α), ultimately leading to reduced protein synthesis [53]. Deletion of PERK has been associated with poor β-cell survival and neonatal diabetes [53,90].

### 3.3. Acetylation

Reversible protein lysine acetylation is under the control of “writer” and “eraser” enzymes, namely the lysine acetyltransferases and deacetylases. In addition to the zinc-dependent lysine deacetylases, the NAD^+^-dependent lysine deacetylases, or the sirtuins, may also contribute to the pathogenesis of T2D [91]. Aberrant hepatic glucose uptake (HGU) is a hallmark of T2D and has been closely associated with declined cellular NAD^+^ levels. Sirt2, one of the mammalian sirtuins, was suggested to be involved in HGU regulation in an NAD^+^-dependent manner. At the molecular level, Sirt2 deacetylates K126 of glucokinase regulatory protein (GKRP) to promote the glucose-dependent dissociation and activation of glucokinase [54], the enzyme that catalyzes the very first step of glycolysis to convert glucose to glucose-6-phosphate. Decreased NAD^+^ contents in the hepatocytes from *db*/*db* mice or high-fat diet (HFD)-fed obese diabetic mice lead to elevated acetylation levels of K126 of GKRP and impaired HGU, presumably through the inhibition of Sirt2 activity [54]. Similarly, hepatic Sirt2 knockdown has also been shown to impair HGU and glucose tolerance in non-diabetic mice. These results indicate the key role the NAD^+^/Sirt2 axis plays in regulating GKRP acetylation and HGU.

Ten–eleven translocation methylcytosine dioxygenase 1 (TET1) is involved in the active demethylation of 5-methylcytosine (5mC) in a recently identified epigenetic reprogramming pathway [92]. The role of TET1 in regulating hepatic glucose metabolism has also been investigated. It was found that TET1 physically interacts with SIRT1 and activates its deacetylase activity on PGC-1a and FoxO1 [55]. The decreased acetylation levels of PGC-1a and FoxO1 promote their nuclear translocation for improved transcription of hepatic gluconeogenic genes. TET1 deficiency, on the other hand, disrupts hepatic glucose homeostasis. Therefore, TET1 has been suggested as a key player in regulating hepatic glucose metabolism and as a potential therapeutic target for the treatment of metabolic diseases such as T2D [55].

### 3.4. SUMOylation

SUMOylation plays an important role in governing the secretory function of β cells. Increasing levels of SUMOylation have been shown to decrease the secretory activity and exocytosis of b cells [93,94]. The SUMOylation of peroxisome proliferator-activated receptor gamma (PPARg) significantly impacts its transcriptional activity. It has been demonstrated that excessive SUMOylation of PPARg, a mimic of a high-fat diet, leads to endothelial insulin resistance and dysfunction through the downregulation of the endothelial nitric oxide synthase–nitric oxide (eNOS-NO) pathway [56]. The over-SUMOylation of PPARg also initiates an endogenous SUMOylation cascade to further worsen the endothelial insulin resistance. In another vein, K107 SUMOylation of PPARg is known to markedly suppress its transcriptional activity [95]. In mice, inhibition of K107 SUMOylation improves insulin sensitivity without inducing adiposity [57]. The effect of PPARg SUMOylation on its transactivation was also investigated. In human umbilical vascular endothelial cells (HUVECs), the upregulation of PPARg SUMOylation and nuclear receptor corepressor (NcoR) expression have been detected in response to high glucose and palmitic acid treatment [58]. This PTM further enhances PPARg and NcoR association, ultimately resulting in endothelial insulin resistance [58].

Gli-similar 3 (Glis3), a transcription factor, is critical in b-cell development and proliferation, as well as the regulation of insulin gene expression [96,97]. Several single-nucleotide polymorphisms (SNPs) in Glis3 have been closely associated with T2D [98]. The transactivation of Glis3 can be modulated by protein inhibitor of activated STAT Y (PIASy) and ubiquitin-conjugating enzyme 9 (Ubc9), two SUMO pathway-related proteins, in a SUMOylation-dependent manner [59]. Notably, at K224 and K430, Glis3 SUMOylation leads to a significant reduction in insulin transcription. Interestingly, chronic hyperglycemia also results in over-SUMOylation of Glis3 for decreased insulin transcription [59].

### 3.5. Oxidation

Increasing evidence suggests that even in healthy β cells, there is a modest degree of proinsulin disulfide mispairing [99,100]. This process is further enhanced by the disruption of ER folding machinery. It has been reported recently that in two murine models of diabetes, the accumulation of misfolded proinsulin complexes due to disulfide mispairing occurs in the early stage of T2D [60]. Protein disulfide isomerase A1 (PDIA1) plays a key role in maintaining proper insulin maturation under metabolic stress through its direct interaction with proinsulin to influence disulfide bond formation [101].

### 3.6. AGEs

AGEs are a group of complex PTMs formed through a series of non-enzymatic reactions between the reducing sugars and the free amino group of the proteins [34]. Hyperglycemia fosters the formation of AGEs, which have been implicated in the pathophysiology of T2D and its related complications [102]. Growing evidence suggests that AGEs exert their effects via either the trapping or crosslinking of proteins or through the binding and activation of receptor proteins [103]. In healthy rats, AGE-albumin treatment induces whole-body insulin resistance and reduced glucose transporter GLUT4 expression in skeletal muscle, among other effects [61]. Mechanistically, AGE-albumin interacts with the receptor for AGEs (RAGE) to activate the NF-*κ*B inflammatory pathway, ultimately leading to the repression of GLUT4 expression [61]. Decreased GLUT4 expression has been observed in mouse models of T2D and T2D patients [104], further highlighting the key role AGEs play in inducing insulin resistance.

## 4. Clinical Trials of Diabetes by Targeting PTMs

Given the importance of PTMs in diabetes, it is not surprising that small molecules targeting PTMs are being investigated in clinical studies as potential therapeutic interventions for T1D and T2D. Some recent clinical trials are summarized in Table 3.

The safety and efficacy of a combination of bempedoic acid (BA) and ezetimibe (EZE) have been evaluated in patients with T2D and hypercholesterolemia [105]. Comparing EZE alone with a placebo, a fixed dosage of 180 mg BA and 10 mg EZE significantly lowered the levels of low-density lipoprotein cholesterol (LDL-C) after 12 weeks, without indication of worsened glycemic control [105]. The mechanism of action of this combination of BA and EZE is two-fold: inhibition of ATP-citrate lyase (ACL) to reduce cholesterol biosynthesis and activation of AMPK to maintain glucose and lipid homeostasis [113].

Curcumin, a polyphenol phytochemical isolated from *Curcuma longa*, has been shown to influence both the AMPK/ACC and *O*-GlcNAcylation pathways [114,115]. In a randomized, double-blinded, placebo-controlled trial in a prediabetic population, 9-month curcumin treatment markedly delayed the onset of T2D, with improved β-cell functions compared to the placebo group [106].

GK activators such as AZD1656 and dorzagliatin have also been investigated in clinical studies for T2D patients. Short-term treatment with AZD1656 was able to reduce HbA1c levels [107]. When combined with metformin, AZD1656 improved glycemic control up to 4 months [108]. Dorzagliatin is a dual-acting GK allosteric activator [116]. In a monotherapy study, dorzagliatin demonstrated improved glycemic control and β-cell functions [109]. In another clinical trial, dorzagliatin showed good glycemic control in combination with metformin [110].

Sodium phenylbutyrate (NaPB), a known HDAC inhibitor, has also been examined for its effect as a branched-chain amino acid (BCAA) catabolism accelerator. NaPB acts as an allosteric inhibitor of the branched-chain ketoacid dehydrogenase (BCKD) kinase, increasing the rate of oxidation of BCAA [117]. In a randomized, double-blind, placebo-controlled study of patients with well-controlled T2D, an NaPB add-on treatment of 4.8 g/m^2^/day over 2 weeks resulted in a 27% increase in peripheral insulin sensitivity, as well as decreases in plasma BCAA and glucose levels [111].

TTP399 acts as a selective hepatic GK activator without affecting the interactions between GK and GKRP [118]. TTP399 was examined as an adjunctive therapy to insulin in T1D patients. TTP399 significantly lowered HbA1C as an insulin adjuvant therapy, with reduced hypoglycemia and a favorable safety profile [112].

Evidently, there is a scarcity of small molecules in ongoing clinical investigations as therapeutics targeting PTMs in diabetes. Additionally, some compounds, such as curcumin, may exhibit broader and off-target effects *in vivo* [119]. The need for an innovative drug design approach is apparent. For example, targeted PTMs by chemically induced proximity (CIP) has emerged as a powerful tool for the modification of a specific PTM [120]. CIPs such as PROTAC [121], DUBTAC [122], PhosTAC [123], and AceTAC [124] are heterobifunctional small molecules capable of bringing the protein of interest (POI) and the post-translational modification enzyme into close proximity for the subsequent covalent modifications.

## 5. Conclusions and Perspectives

Protein PTMs alter protein function, stability, and subcellular localization, as well as protein–protein interactions (PPIs). They are essential for maintaining normal physiology but, when dysregulated, can lead to diseases such as diabetes. In this review, we summarize the current progress pertaining to PTMs involved in T1D and T2D. A broad array of PTMs have been implicated in the initiation and progression of diabetes, regulating molecule transport, immune response, signaling cascade, cell function, and metabolite homeostasis. Therefore, these PTMs can serve as potential biomarkers or therapeutic targets for diabetes.

Currently, metformin continues to be the first-line medication for T2D, with thiazolidinediones (TZDs), GLP-1 receptor agonists, dipeptidyl peptidase 4 (DPP-4) inhibitors, and sodium-glucose cotransporter-2 (SGLT2) inhibitors serving as alternative medications. In addition to their roles in the pathogenesis of diabetes, PTMs can also impact the efficacy of these diabetic medications. For example, SUMOylation of GLP-1 receptor has been shown to decrease incretin responsiveness [93], underlying the reduced clinical efficacy of GLP-1 receptor agonists in certain patient populations. In the same vein, TZDs are associated with well-known side effects such as weight gain [125]. Blocking SUMOylation of PPARg, the target of TZDs, enhances insulin sensitivity without triggering weight gain [57]. Hence, deciphering the mechanism of PTM regulation is beneficial in improving existing medications.

In addition to the small-molecule approach, gene editing technology has also been explored for the treatment of diabetes. A recent study demonstrated the correction of a diabetes-causing gene variant in Wolfram syndrome 1 (*WFS1*) using CRISPR-Cas9 in induced pluripotent stem cells (iPSCs) [126]. The corrected iPSCs differentiate into SC-β cells with improved insulin secretion capability in response to glucose *in vitro*. The transplantation of these gene-corrected β cells reverses hyperglycemia in diabetic mice [126]. This study may pave the way for the development of autologous cell replacement therapy for diabetic patients.

Owing to the rapid advancements in proteomics techniques, more than 600 PTMs have been identified in human proteomes [127]. Each PTM alters protein function in its unique way in response to cellular and environmental stimuli. Many proteins are differentially controlled by multiple PTMs, and the crosstalk between PTMs has emerged as a highly relevant regulatory mechanism. For example, phosphorylation of PPARγ2 at Ser112 enhances the SUMOylation at K107 to negatively regulate the transactivation of PPARγ2 [128]. A deeper understanding of the complex interplay between PTMs in the context of diabetes is critical for identifying a potential regulatory mechanism and therapeutic targets.

In summary, PTMs have profound functional consequences in various cellular events associated with diabetes. Therefore, investigation of PTMs not only is critical for unraveling the pathophysiology of diabetes but also offers unprecedented opportunities to advance the management and treatment of diabetes and related complications.

## Figures and Tables

**Table 1:** PTMs in T1D.

PTM	Proteins	Function	Reference
Oxidation	Insulin	Antibodies to oxidized insulin (oxPTM-INS-Ab) improve T1D risk assessment	[21]
	HSA	Initiation and progression of T1D	[22]
Glycation	lgG	Decreased IgG function	[23]
*N*-Glycosylation	Plasma proteins, IgG, and C3	Risk factor for early-onset T1D	[24,25]
Carbonylation	P4Hb	Decreases glucose-stimulated insulin secretion and alters proinsulin-to-insulin ratios	[26]
Citrullination	GK	Impairs islet response to glucose and overall glucose homeostasis	[27]
Deamidation	Proteins in NOD mice and pancreatic islets	Generation of deamidated autoantigens in T1D	[28]
*O*-GlcNAcylation	c-Rel	Reduces immunosuppressive FOXP3 expression	[29]
	HDAC4	Enhances the production of the cardio-protective *N* terminal of HDAC4	[30]
SUMOylation	IRF4	Promotes macrophage M2 polarization and energy homeostasis	[31]
Methylation	Histone H3	Suppresses GLUT4 expression and worsens glycemic impairment	[32]

**Table 2:** PTMs in T2D.

PTM	Proteins	Function	Reference
*O*-GlcNAcylation	p53	Activates gluconeogenic PCK1 transcription	[42]
	Sp1	Downregulation of GTSP expression for the development of oxidative stress	[43]
	SIRT1	Controls liver metabolic switching and hyperglycemia prevention	[44]
	eIF4G1	reverses hyperproinsulinemia	[45]
	SERCA2	Restores insulin secretion	[46]
	PLIN1	Retains fat mass in adipose tissue, triggers diet-induced obesity, and leads to whole-body insulin resistance	[47]
Phosphorylation	PKA and PKC	Hyperglycemia	[48]
	PDX1	Impairment of β-cell functions	[48]
	VEGFR2	Regulates brown adipocyte differentiation	[49]
	NMDAR	Results in β-cell hyperpolarization and a reduction in glucose-mediated insulin secretion	[50]
	FoxO1	Insulin resistance	[51]
	FDAA	Insulin degradation	[52]
	eIF2α	Reduced protein synthesis	[53]
Acetylation	GKRP	Impairment of hepatic glucose uptake	[54]
	PGC-1α and FoxO1	Regulates hepatic glucose homeostasis	[55]
SUMOylation	PPARγ	Endothelial insulin resistance	[56,57,58]
	Glis3	Reduction in insulin transcription	[59]
Oxidation	Proinsulin	Proinsulin misfolding, ER stress, and β-cell failure	[60]
AGEs	Albumin	Insulin resistance and decreased GLUT4 expression	[61]

**Table 3:** Clinical trials of diabetes by targeting PTMs.

Drug	Disease	Clinical Trial ID	PTM	Outcome	Reference
Bempedoic acid/Ezetimibe	T2D	NCT03531905	Phosphorylation	Lowered low-density lipoprotein cholesterol and improved high-sensitivity C-reactive protein	[105]
Curcumin	T2D	NCT01052025	Phosphorylation Ubiquitination *O*-GlcNAcylation	Delayed onset of T2D from prediabetes	[106]
AZD1656	T2D	NCT01152385 NCT01020123	*S*-nitrosylation SUMOylation	Reduction in HbA1C after short-term treatment; Improvement of glycemic control in combination with metformin up to 4 months	[107,108]
Dorzagliatin	T2D	NCT02561338 NCT03141073	*S*-nitrosylation SUMOylation	Beneficial effect on glycemic control; Effective glycemic control in combination with metformin	[109,110]
Sodium phenylbutyrate	T2D	NTR7426	Acetylation	Increased peripheral insulin sensitivity and reduced plasma branched-chain amino acids and glucose levels	[111]
TTP399	T1D	NCT03335371	*S*-nitrosylation SUMOylation	Lowered HbA1C and reduced hypoglycemia	[112]
